# A Sensitivity Analysis-Based Parameter Optimization Framework for 3D Printing of Continuous Carbon Fiber/Epoxy Composites

**DOI:** 10.3390/ma12233961

**Published:** 2019-11-29

**Authors:** Hong Xiao, Wei Han, Yueke Ming, Zhongqiu Ding, Yugang Duan

**Affiliations:** State Key Lab for Manufacturing Systems Engineering, Xi’an Jiaotong University, Xi’an 710049, China; jx18hanwei@xjtu.edu.cn (W.H.); mingyueke@stu.xjtu.edu.cn (Y.M.); dingzhq@stu.xjtu.edu.cn (Z.D.); ygduan@xjtu.edu.cn (Y.D.)

**Keywords:** continuous carbon fiber/epoxy composites, 3D printing, parameter optimization, sensitivity analysis

## Abstract

Three-dimensional printing of continuous carbon fiber/epoxy composites (CCF/EPCs) is an emerging additive manufacturing technology for fiber-reinforced polymer composites and has wide application prospects. However, the 3D printing parameters and their relationship with the mechanical properties of the final printed samples have not been fully investigated in a computational and quantifiable way. This paper presents a sensitivity analysis (SA)-based parameter optimization framework for the 3D printing of CCF/EPCs. A surrogate model for a process parameter–mechanical property relationship was established by support vector regression (SVR) analysis of the experimental data on flexural strength and flexural modulus under different process parameters. An SA was then performed on the SVR surrogate model to calculate the importance of each individual 3D printing parameter on the mechanical properties of the printed samples. Based on the SA results, the optimal 3D printing parameters and the corresponding flexural strength and flexural modulus of the printed samples were predicted and verified by experiments. The results showed that the proposed framework can serve as a high-accuracy tool to optimize the 3D printing parameters for the additive manufacturing of CCF/EPCs.

## 1. Introduction

Fiber-reinforced polymer composites (FRPCs) have been widely used in aerospace, transportation, and construction industries due to the fact of their low density, outstanding designability, and high strength and modulus [1,2]. However, complex molds, multiple preparation steps, long production cycles, and high manufacturing costs are typically required in conventional manufacturing processes of FRPCs including hand paste molding, resin transfer molding, and filament winding [3,4]. Three-dimensional printing is a layer-by-layer additive manufacturing technology with no mold, high speed, and low cost [5,6,7]. Therefore, using 3D printing to develop an automated, low-cost, and moldless manufacturing process for FRPCs can promote further development and application of these materials [8,9,10].

Recently, it has been reported that the 3D printing of FRPCs has achieved remarkable milestones, from thermoplastics to thermosetting polymers and from short fibers to continuous fibers [11,12,13,14]. Initially, short fibers were added into the thermoplastic matrix of 3D printing materials (in most cases, acrylonitrile butadiene styrene (ABS) or polylactic acid (PLA)) to increase the tensile and flexural strengths. For example, Tekinalp et al. [15] and Ning et al. [16] prepared short carbon fiber (SCF)-reinforced ABS composite filament and printed it using fused filament fabrication (FFF) equipment. The 3D printed SCF/ABS samples with 40 wt% fiber content exhibited a tensile strength and modulus of 65.0 MPa and 13.6 GPa, respectively, while those of the ABS samples without SCF exhibited 31 MPa and 2.2 GPa, respectively. Due to the weak load carrying capacity, poor interlayer bonding, and low strength and hardness of the thermoplastic matrix, Compton et al. [5] replaced it with thermosetting epoxy resin (EP) and an imidazole compound hardener. Benefiting from the irreversible chemical bonds formed after curing, the tensile strength of the 3D printed SCF/EP samples with 35 wt% fiber content increased to 66.2 MPa from 56.9 MPa for the EP samples without SCF. After achieving partial strength improvement by adding SCF, researchers found that continuous fibers could provide a much stronger mechanical response due to the fiber continuity which could be used for 3D printing to further improve the mechanical properties [12,17,18]. For example, Matsuzaki et al. [19] and Tian et al. [20] introduced continuous carbon fiber (CCF) into PLA and conducted 3D printing through a multi-channel printing head. The tensile and flexural strengths of the 3D printed CCF/PLA samples with 27 wt% fiber content increased significantly to 220 MPa and 335 MPa, respectively, from 60 MPa for the PLA samples without CCF. However, the low interlaminar shear strength of 2.81 MPa [11] illustrated the weak interlayer bonding due to the thermoplastic matrix. Therefore, Hao et al. [21] and Ming et al. [13] used molten EP to impregnate CCF and the pre-formed samples were subjected to a thermal post-curing process after printing. The cured CCF/EP samples with 48 wt% fiber content exhibited the tensile and flexural strengths of 792.8 MPa and 202.0 MPa, respectively, while those of the EP samples without CCF were 56.9 MPa and 103 MPa, respectively. However, the detailed process parameters and their relationship with the mechanical properties of the final printed samples have not yet been discussed.

Generally, process parameters, such as printing speed, temperature, and pressure, have relatively large ranges. A slight variation may cause a significant change in the final mechanical properties [22]. Although the experimental method would be a preferred and reliable way to acquire the optimal parameters, it is practically difficult or even impossible to carry out sufficient experiments because it would be a time-consuming and unaffordable process. Sensitivity analysis (SA) evaluates how the variations in the model output can be apportioned to variations in model inputs [23]. It is widely used in various disciplines to determine the key input variations that have great influence on the model output. Lurette et al. [24] adopted the SA method to identify key parameters influencing salmonella infection in a pig batch. Makowski et al. [25] used SA to calculate the contribution of genetic parameters to the variance of crop model prediction. Saltelli et al. [26] determined the strength of the relationship between a given uncertain input and the output based on SA. In addition to being used in biology and chemistry, SA has also been applied in engineering [27] and environmental science [28].

**Figure 1:**
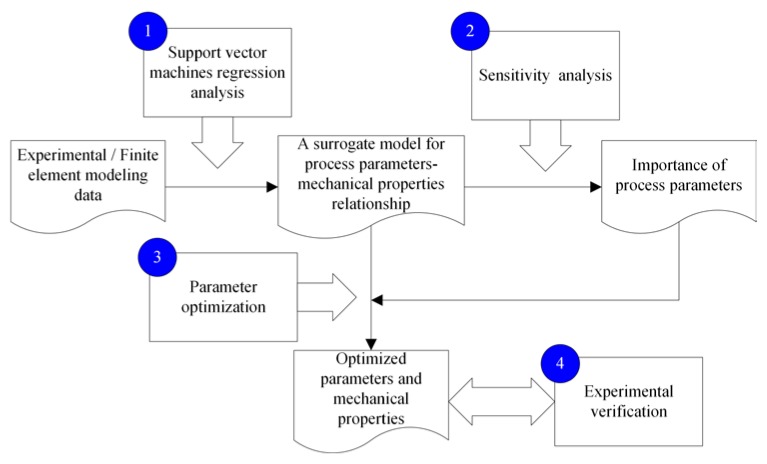
The outline of the proposed framework.

This paper reports an SA-based parameter optimization framework for the 3D printing of CCF/EP composites (CCF/EPCs). The outline is illustrated in Figure 1. Firstly, the experimental data on flexural strength and modulus under different process parameters were analyzed, and a surrogate model for a process parameter–mechanical property relationship was established. Then, the importance of the process parameters was calculated using the SA approach. Subsequently, the optimal process parameters and the corresponding mechanical properties were calculated based on the SA results. Finally, an experiment with the simulated optimized process parameters was conducted to verify the proposed SA-based process optimization framework.

## 2. Experimental Setup and Data Validation

### 2.1. Experimental Setup

#### 2.1.1. Raw Materials for 3D Printing

For reinforcement, 3K polyacrylonitrile (PAN)-based carbon fibers (3000 fibers in a bundle, Tenax^®^-J, HTS40, 200 tex, Toho Tenax, Co., Ltd., Tokyo, Japan) were used. The thermosetting matrix was composed of an epoxy resin (D.E.R. 671 (EP-671), 95 wt%, Dow, Pittsburg, CA, USA) and a thermally induced latent hardener (dicyandiamide (DICY), 5 wt%, Yongxin Plasticization, Guangzhou, China).

#### 2.1.2. 3D Printing Process and Mechanical Property Test

**Figure 2:**
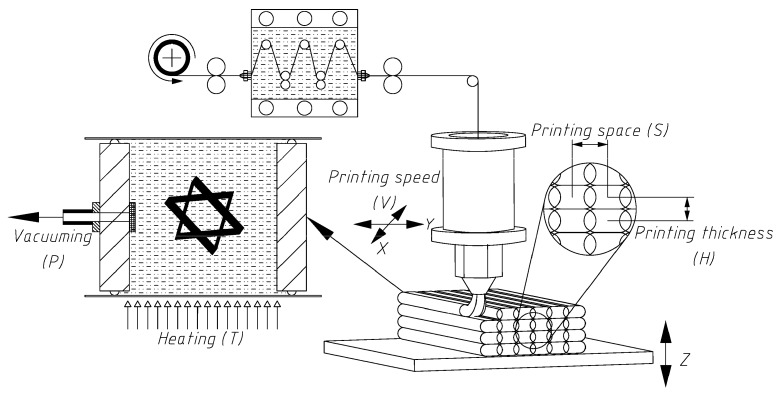
The 3D printing process for the continuous carbon fiber/epoxy composites (CCF/EPCs).

Figure 2 shows the three main steps of the 3D printing process of the CCF/EPCs. First, the 3K carbon fibers were conveyed into the molten resin tank (130 °C) to impregnate EP-671 at a low viscosity. Then, the impregnated filament was fed to the printing head for subsequent printing. After extrusion from the printing nozzle, the printed filament was rapidly cooled and solidified to attach to the substrate. After that, the pre-formed samples were cured at a high temperature and in a vacuum environment.

The 3D printing process for the CCF/EPCs is simple and straightforward. Nevertheless, it requires a lot of effort to address the challenge of controlling the parameters during the 3D printing process. In this process, the critical parameters are printing speed, printing space, and printing thickness during the printing phase and the curing temperature and curing pressure in the curing phase. Printing speed (*V*) is defined as the speed of the printing nozzle moving in the X–Y plane. Printing speed (*V*) determines the feeding and conveying speed of the impregnated fibers which affects the extensional flow of the molten resin matrix and, consequently, the mechanical properties of the printed parts. Printing space (*S*) is defined as the central distance between two adjacent printed fiber bundles which should have certain overlap by controlling *S* to strengthen the bonding of the adjacent fibers and avoid gap defects of the printed parts. Similarly, certain overlap among two adjacent layers can strengthen the bonding and yield printed parts with improved mechanical properties. The adjacent layer overlap can be controlled by adjusting the printing platform along the *z*-axis for a suitable printing thickness (*H*) which is the central distance between two adjacent printed layers. Curing temperature (*T*) and curing pressure (*P*) in the curing phase are the parameters that affect the viscosity and flow of the molten resin matrix and, eventually, influence the void ratio of the printed samples.

For the 3D printer and the printing materials used in this work, the set of process parameters is listed in Table 1.

The standard CCF/EPC testing samples with the dimensions of 100 mm × 15 mm × 2 mm were printed on the 3D printer prototype and subsequently cured. Then, three-point bending tests were conducted using an electromechanical universal testing machine (MTS systems, Co., Ltd., Shenzhen, China) to measure the flexural strength and modulus of the 3D printed samples according to ISO 14125 standard (fiber-reinforced plastic composites–determination of flexural properties).

### 2.2. Experimental Data Validation

**Figure 3:**
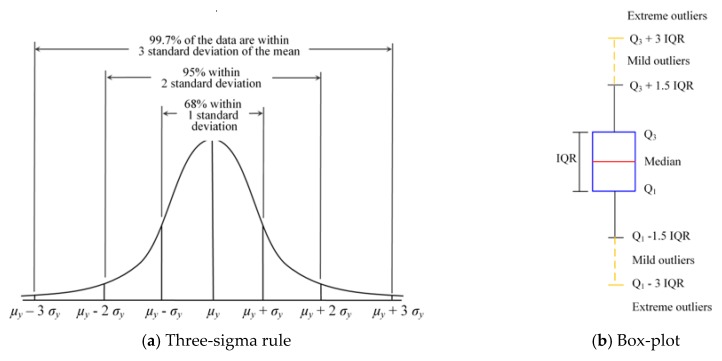
(**a**) Three-sigma rule and (**b**) box-plot.

Due to the variability in the measurement or experimental error, outliers may be present in the experimental data which can cause serious problems in the analysis. Therefore, two methods were employed in this paper to cross validate the experimental data: the well-known three-sigma rule [29] and box-plot [30] (Figure 3). Only if an observation was identified as an outlier by both methods was it excluded from the dataset in the subsequent analysis.

#### 2.2.1. Three-Sigma Rule

The three-sigma rule is a simple and commonly used criterion for detecting outliers. For a set of experimental data {*y*_1_, *y*_2_, …, *y*_N_}, their mean value and standard deviation are denoted by *µ_y_* and *σ_y_*, respectively. Assuming that this set of data follows a normal distribution, the three-sigma rule indicates that the probability of a point falling outside the interval [*µ_y_* – 3 *σ_y_*, *µ_y_* + 3 *σ_y_*] is only 0.27%. Therefore, if a datum point falls outside the interval [*µ_y_* – 3 *σ_y_*, *µ_y_* + 3 *σ_y_*], it is considered an outlier which contains a gross error and should be removed from the dataset. It is worth noting that the outliers in the data should be removed one at a time. Once a point is excluded from the dataset, the sample size becomes *N* = *N* − 1. Then, the new three-sigma interval is recomputed from the remaining *N* − 1 data, based on which the new outlier is detected. This process is repeated until all the outliers are identified. Three-sigma rule depends on the mean value and standard deviation of the dataset which may be influenced by the outliers. Therefore, the box-plot was adopted to cross validate the experimental data.

#### 2.2.2. Box-Plot

The box-plot is based on the interquartile range (IQR) of the dataset. Assuming that Q_1_ and Q_3_ are the lower and upper quartiles of the dataset, respectively, an outlier can be defined as any observation outside the range [Q_1_ – *k* IQR, Q_3_ + *k* IQR], where IQR = Q_3_ – Q_1_. Tukey [30] proposed in his work that *k* = 1.5 indicates a “Mild outlier”, and *k* = 3 suggests the data are an “Extreme outlier”. Thus, this method is also known as Tukey’s fences. In this paper, *k* = 3 was chosen to retain as many experimental data as possible. Similar to the three-sigma rule, the outliers in the box-plot method are also detected one at a time. Once an observation is excluded from the dataset, the lower and upper quartiles, Q_1_ and Q_3_, are searched again, and the interval for detecting outliers is recomputed. This process is repeated until all the outliers are identified. Since Q_1_ and Q_3_ only rely on the order of the data, the box-plot method is less influenced by the outliers.

## 3. Regression Analysis for a Surrogate Model of a Process Parameter–Mechanical Property Relationship

Support vector machines have been widely used for classification (support vector classification, SVC) and regression (support vector regression, SVR) purposes, as they are highly competitive in terms of accuracy compared with other classification and regression methods, especially when dealing with a small sample dataset [31].

For the regression problem in this paper, the training data were ***D*** = {**x***_i_*, *y_i_*}. **x***_i_* ∈ **R***^d^* is a vector composed of the 3D printing parameters described in Section 2. *y_i_* ∈ **R** is the mechanical property (flexural strength or flexural modulus). *i* = 1, ..., *l* is the number of experiment datasets, and *d* is the number of 3D printing parameters. Suppose the training data can be fitted by a function *f*(**x**):

*f*(**x**) = *ω*·*φ*(**x**) + *b* (1)

where *φ*(**x**) maps the **x** to a higher dimensional space as the data are non-linear; *ω* and *b* are the coefficients. The fitting or surrogate modelling problem in SVR is to seek a small *ω* [32], i.e.,

(2) $$\begin{array}{c} \min\;\frac{1}{2}∥\omega {∥}^{2}\\ s.t.\;\{\begin{array}{c} y_{i}-\omega \cdot \varphi \left(x_{i}\right)-b\leq \varepsilon \\ \omega \cdot \varphi \left(x_{i}\right)+b-y_{i}\leq \varepsilon \end{array} \end{array}$$

where *ε* is the insensitive coefficient, representing the range of acceptable error of the surrogate model. As some of the training data might fall out of the insensitive domain decided by *ε*, slack variables $\xi_{i}$ and $\xi_{i}^{*}$ are introduced to each dataset so that more data can be used for training. The constant *C* > 0 balances the smoothness of function *f*(**x**) and the training error. Thus, the optimization problem in Equation (2) can be described as follows:

(3) $$\begin{array}{c} \min\;\frac{1}{2}∥\omega {∥}^{2}+C\overset{l}{\underset{i\;=1}{\sum }}\left(\xi_{i}+\xi_{i}^{*}\right)\\ s.t.\;\{\begin{array}{c} y_{i}-\omega \cdot \varphi \left(x_{i}\right)-b\leq \varepsilon +\xi_{i}\\ \omega \cdot \varphi \left(x_{i}\right)+b-y_{i}\leq \varepsilon +\xi_{i}^{*}\\ \xi_{i},\;\;\xi_{i}^{*}\;\geq 0 \end{array} \end{array}$$

This problem can be solved more easily in its dual formulation [32]:

(4) $$\begin{array}{c} \begin{array}{c} \min\;\frac{1}{2}\overset{l}{\underset{i,j=1}{\sum }}\left(\alpha_{i}-\alpha_{i}^{*}\right)\left(\alpha_{j}-\alpha_{j}^{*}\right)k\left(x_{i}\cdot x_{j}\right)\\ +\overset{l}{\underset{i=1}{\sum }}\alpha_{i}\left(\varepsilon -y_{i}\right)+\overset{l}{\underset{i=1}{\sum }}\alpha_{i}^{*}\left(\varepsilon +y_{i}\right) \end{array}\\ s.t.\;\;\{\begin{array}{c} \overset{l}{\underset{i=1}{\sum }}\left(\alpha_{i}-\alpha_{i}^{*}\right)=0\\ \alpha_{i},\;\;\alpha_{i}^{*}\in \;\left[0,\;\;C\right] \end{array} \end{array}$$

where $\alpha_{i}$ and $\alpha_{i}^{*}$ are the Lagrange multipliers, and the kernel function $k\left(x_{i}\cdot x_{j}\right)$ is adopted to map the data to higher dimensional space. In this work, Gaussian radial basis function (RBF) $k\left(x_{i}\cdot x_{j}\right)=\exp\left(-{\left|x_{i}-x_{j}\right|}^{2}/2\sigma^{2}\right)$ was used as the kernel function. The parameter *σ* will influence the distribution of the training data in the new higher dimensional space. Then, the fitting function in Equation (1) can be rewritten in support vector expansion, i.e., $=\sum_{i=1}^{l}\left(\alpha_{i}-\alpha_{i}^{*}\right)x_{i}$, which is described as the linear combination of the training data *x_i_*. Thus, $f\left(x\right)=\sum_{i=1}^{l}\left(\alpha_{i}-\alpha_{i}^{*}\right)k\left(x_{i}\cdot x_{j}\right)+b$. The Karush–Kuhn–Tucker (KKT) condition is used to obtain the coefficient *b* [33].

Based on the data acquired in Section 2, the surrogate model of the process parameter–mechanical property relationship can be established by performing the above regression analysis and, subsequently, be used for the SA of the process parameters.

To ensure the practical applicability of the surrogate model, it is necessary to validate the accuracy of the model before prediction. If the accuracy of the surrogate model does not meet the engineering requirement, the model should be improved either by adjusting the model coefficients or increasing the training sample size.

One of the most commonly used errors for assessing the accuracy of the surrogate model is the root mean square error (RSME), which is defined as follows:

(5) $$\mathrm{RSME}=\;\sqrt{\frac{1}{M}\overset{M}{\underset{i=1}{\sum }}{\left(y_{i}-{\hat{y}}_{i}\right)}^{2}}\;$$

where *y_i_* is the *i*th response value obtained from the experiment, *ŷ*_i_ is the corresponding response value predicted by the surrogate model under the same input condition, and *M* is the number of samples used for validation.

However, the validation data from an experiment are often limited due to the experimental cost. Therefore, it is not enough to obtain a comprehensive RSME. A more reliable method to verify the accuracy of the surrogate model is the cross-validation method which randomly divides the experimental and predicted data into several groups, and then the training and validation of the surrogate model are performed with each group in turn. The procedure of the commonly used *k*-fold cross-validation is as follows [34]:

- Step 1: Randomly divide the experimental and the corresponding predicted data into *k* groups;
- Step 2: Leave one group of the data for the validation of the surrogate model accuracy, and train the surrogate model with the data of the remaining *k* − 1 groups;
- Step 3: Repeatedly perform Step 2 *k* times until each group of the data has been used for model validation. Choose the model with the minimum RSME as the final model. The final accuracy of the surrogate model is measured by the mean of all the RSME of the *k*-trained surrogate model.

## 4. Sensitivity Analysis of the Process Parameters

As there are several process parameters, it is important to analyze their influence on the mechanical properties of the printed samples. Sensitivity analysis can identify the input parameters which have a large influence on the model output, and it is generally classified into local SA and global SA [35]. Local SA investigates how much the output is changed by the small variation in the input parameters around a reference point (such as the mean value). The quality of local SA relies on the choice of the reference point. Instead, global SA can measure the effect of each individual input parameter or their interactions on the output of the model within the entire range space of the input. Therefore, global SA is independent of the selection of the reference point. The variance-based SA method, which is one of the most popular global SA methods, was used in this paper and is briefly introduced in this section.

Suppose that the SVR model established in Section 3 is *Y* = g(***X***) with *Y* as scalar output and ***X*** = (*X*_1_, *X*_2_, …, *X_d_*) as input vector. In this paper, *Y* is the flexural strength or flexural modulus of the 3D printed CCF/EPCs samples, and ***X*** represents the input parameters of the 3D printing process, i.e., printing speed, printing space, printing thickness, curing pressure, and curing temperature.

According to the theory of the analysis of the variance [36], the total variance of the output when the input parameters are independent can be decomposed into:

(6) $$Var\;\left(Y\right)=\;\overset{d}{\underset{i=1}{\sum }}V_{i}+\;\overset{d}{\underset{1\leq i\leq j\leq d}{\sum }}V_{ij}+\cdots +\;V_{12\ldots d}$$

where

(7) $$\begin{array}{c} V_{i}=Var\;\left[E\left(Y|X_{i}\right)\right]\\ V_{ij}=Var\;\left[E\left(Y|X_{i},X_{j}\right)\right]-\;V_{i}-V_{j}\\ \ldots \end{array}$$

where *Var* and *E* are the variance and expectation operators, respectively, and *Var*(*Y*) is the total variance of the output *Y*. The variation of *Y* associated with changes in the input variable *X_i_* with no reference to other variables is then given by the single effect index as:

(8) $$S_{i}=\;\frac{V_{i}}{Var\left(Y\right)}=\;\frac{Var\;\left[E(Y|X_{i})\right]}{Var\left(Y\right)}$$

The variation of *Y* caused by variations in input variable *X_i_* interacting with other variables is measured by the sum of all the variances associated with terms where *X_i_* appears. The associated sensitivity index is the total effect index:

(9) $$ST_{i}=\frac{V_{i}+\sum_{1\leq i\neq j\leq d}^{d}V_{ij}+\cdots +V_{12\ldots d}}{Var\left(Y\right)}=1-\frac{Var[E(Y|X_{∼i})]}{Var\left(Y\right)}$$

where $X_{∼i}$ indicates the array of all input variables except *X_i_*.

The single effect index, *S_i_*, provides a reasonable way to rank the importance of the individual input parameter according to its effect on the output. The total effect index, *ST_i_*, summarizes all the effects of *X_i_* on the output, including the single effect and all the other effects caused by the interaction between *X_i_* and $X_{∼i}$ [37]. Therefore, the difference between *S_i_* and *ST_i_* measures the influence on the output by the interaction between *X_i_* and $X_{∼i}$. When *ST_i_* is very small, the corresponding input parameter *X_i_* can be considered as an irrelevant parameter which can be constrained to an arbitrary value within its range without significant effect on the variation of the target output.

In this paper, both the single effect index, *S_i_*, and the total effect index, *ST_i_*, were employed to perform global SA for the CCF/EPC samples. The main purpose was to assess the influence of the input parameters and their interactions on the flexural strength or flexural modulus of the composites.

## 5. Optimization of the 3D Printing Parameters of CCF/EPCs

The optimal 3D printing parameters of CCF/EPCs should be the ones which maximize the flexural strength or flexural modulus of the printed CCF/EPCs. Suppose that the important parameters identified by the SA are denoted by $X^{*}=\left(X_{1}^{*},X_{2}^{*},\ldots ,X_{m}^{*}\right)$, and the corresponding irrelevant parameters are represented by ${\bar{X}}^{*}=\left({\bar{X}}_{1}^{*},{\bar{X}}_{2}^{*},\ldots ,X_{d-m}^{*}\right)$, where $X^{*}\cup {\bar{X}}^{*}=X$ and $X^{*}\cap {\bar{X}}^{*}=\;\emptyset$. The optimization model of the composite can be expressed as follows:

(10) $$\begin{array}{c} \max Y=g\left(X\right)\\ s.t.X_{j}^{*}\in \left[X_{j}^{L*},X_{j}^{U*}\right],j=1,\ldots m\\ {\bar{X}}_{k}^{*}={\bar{x}}_{k}^{*},k=1,\ldots d-m \end{array}$$

where $X_{j}^{L*}$ and $X_{j}^{U*}$ are the lower and upper bounds of the important parameters $X_{j}^{*}$, and ${\bar{x}}_{k}^{*}$ is any value in the range of the irrelevant parameters ${\bar{X}}_{k}^{*}$. The optimization model in Equation (10) searches the maximum of the flexural strength or flexural modulus in the entire range of the important input parameters when all the parameters are varied simultaneously. Thus, it can consider both the individual variation and interactions of the input parameters in the optimization process.

## 6. Results and Discussion

In this section, the proposed framework was applied to analyze the influence of the input parameters on the flexural strength and the flexural modulus of CCF/EPCs and to identify the optimal parameters for the 3D printing of CCF/EPCs. The results for the flexural strength and flexural modulus can cross validate each other and demonstrate the effectiveness of the proposed framework.

### 6.1. Experimental Data Validation

The experimental data are listed in Table 2. The first 25 rows show the adopted 3D printing parameters and the corresponding flexural strength and flexural modulus of the 3D printed samples by the L_25_(5^6^) orthogonal experimental design. The remaining 21 rows are the inputs and outputs of the additional experiment by the single-factor variable control method to enhance the experimental data for the training purpose.

**Figure 4:**
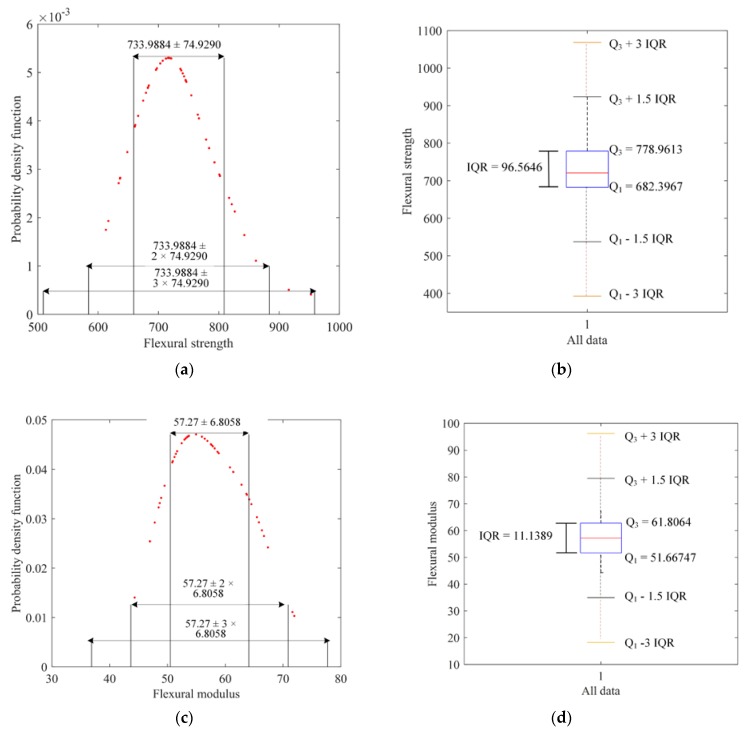
Validation of the experimental data: the distribution of all data under the three-sigma rule (**a**) for flexural strength and (**c**) for flexural modulus and the box-plot form (**b**) for flexural strength and (**d**) for flexural modulus.

First, the experimental data were examined with the three-sigma rule. The mean value and standard deviation of the experimental data of the flexural strength were 733.9884 and 74.9290, respectively. According to the three-sigma rule, the first outlier of the experimental data of flexural strength should be smaller than 509.2013 or larger than 958.7754. As can be seen, there was no outlier in the experimental data of flexural strength according to the three-sigma rule (Figure 4a).

Then, these experimental data of flexural strength were validated according to the box-plot criterion. The first outlier should be the one which falls outside the interval [392.7029, 1068.7]. It can be seen that there was no outlier in the experimental data of flexural strength (Figure 4b). Therefore, all the data of flexural strength obtained in the experiment were considered valid and were used in the subsequent analysis.

Similarly, both the three-sigma rule and box-plot criterion indicate that there was no outlier in the experimental data of flexural modulus, as shown in Figure 4c,d.

### 6.2. Construction of the SVR Surrogate Model

Based on the experimental data of the five input parameters of the composites and the corresponding response values (i.e., flexural strength and flexural modulus given in Table 2), the SVR model of the process parameter–mechanical property relationship was constructed in MATLAB R2017a, and Gaussian RBF was selected which yields the minimum RMSE. The data in Table 2 were randomly divided into 10 groups, and the SVR model was trained and tested by cross-validation. The SVR model used for the prediction of flexural strength was the one with the minimum RMSE 23.6299, i.e., 3.22% error relative to the expectation of the data of flexural strength. The mean RMSE of the 10 SVR models of the process parameter–flexural strength relationship was 58.0936 which indicates a 7.91% mean error relative to the expectation of the data of flexural strength. The SVR model employed for predicting the flexural modulus was the one with the minimum RMSE 2.5921, i.e., a 4.53% error relative to the expectation of the experimental data of flexural modulus. The mean RMSE of the 10 SVR models of the process parameter–flexural modulus relationship was 4.7019 which indicates an 8.21% mean error relative to the expectation of the data of flexural modulus. Therefore, both the SVR models meet the needs of the analysis.

### 6.3. SVR Model-Based SA of the 3D Printing Parameters of CCF/EPCs

**Figure 5:**
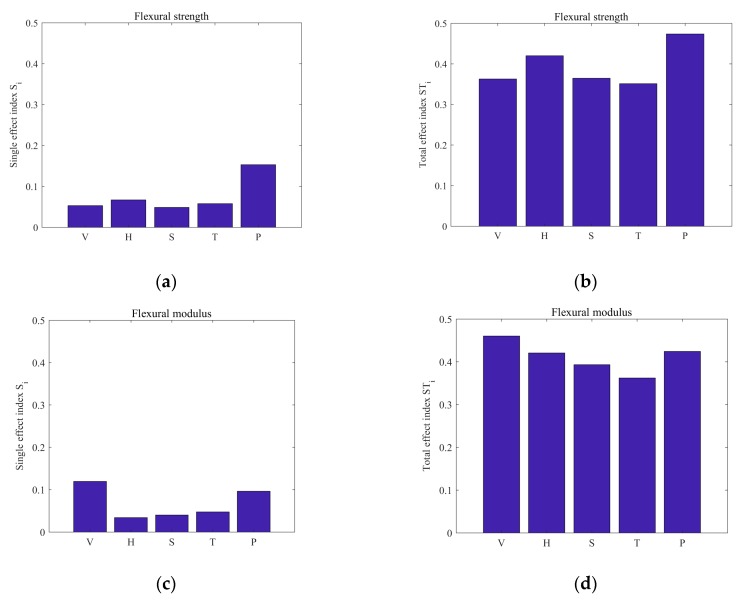
The sensitivity analysis (SA) results of the 3D printing parameters with respect to the flexural strength (**a**,**b**) and flexural modulus (**c**,**d**) of the CCF/EPCs.

Using the Design of Experiments (DOE) tool box in MATLAB, Latin hypercube sampling (LHS) was carried out with the sample size of 10^4^ for the input parameters (printing speed *V*, printing space *S*, printing thickness *H*, curing pressure *P*, and curing temperature *T*). Then, SA was performed based on the constructed SVR model. Figure 5 shows the single effect index, *S_i_*, and the total effect index, *ST_i_*, of the five input parameters with respect to the flexural strength and flexural modulus of CCF/EPCs. It can be seen that the most important parameter that influenced the variation of the flexural strength was the curing pressure *P*, and all the other input parameters had almost equivalent importance for the flexural strength (Figure 5a). For the flexural modulus, the printing speed, *V*, was the most important parameter, followed by the curing pressure, *P*, with the rest of the parameters having nearly equivalent importance (Figure 5c). For both the flexural strength and flexural modulus, the single effect indices, *S_i_*, of all the input parameters were small, and the total effect indices, *ST_i_*, of the input parameters were significantly larger than the corresponding single effect indices, *S_i_* (Figure 5b,d). This indicates that change of an individual input parameter actually had a minor effect on the variation of the flexural strength and flexural modulus which were mainly affected by the interactions among the input parameters. Therefore, the existing optimization methods which consider the variation of one input parameter at a time with all the other parameters being fixed are not appropriate for identifying the optimal 3D printing parameters of the CCF/EPCs.

### 6.4. 3D Printing Parameter Optimization of CCF/EPCs

According to the SA results in Section 6.3, the total effect indices of all the input parameters were large, indicating that all the input parameters had relevant effects on the flexural strength and flexural modulus of the 3D printed CCF/EPCs and, thus, should be considered in the optimization process. The specific optimization model for the 3D printing of CCF/EPCs can be expressed as follows:

(11) $$\begin{array}{c} \min\;-\sigma_{\max}\;\;\mathrm{or}\;-E_{\max}\\ \begin{array}{c} \begin{array}{c} s.t.\;\;V\in \;\left[200,\;\;\;1400\right]\;\mathrm{mm}/\min\\ H\in \;\left[0.25,\;\;\;0.45\right]\;\mathrm{mm} \end{array}\\ \begin{array}{c} S\in \;\left[1,\;\;\;1.4\right]\;\mathrm{mm}\\ T\in \;\left[150,\;\;\;190\right]\;{}^{\circ}C\\ P\in \;\left[-100,\;\;-20\right]\;\mathrm{kPa} \end{array} \end{array} \end{array}$$

where *σ*_max_ and *E*_max_ are the maximum flexural strength and flexural modulus, respectively.

**Figure 6:**
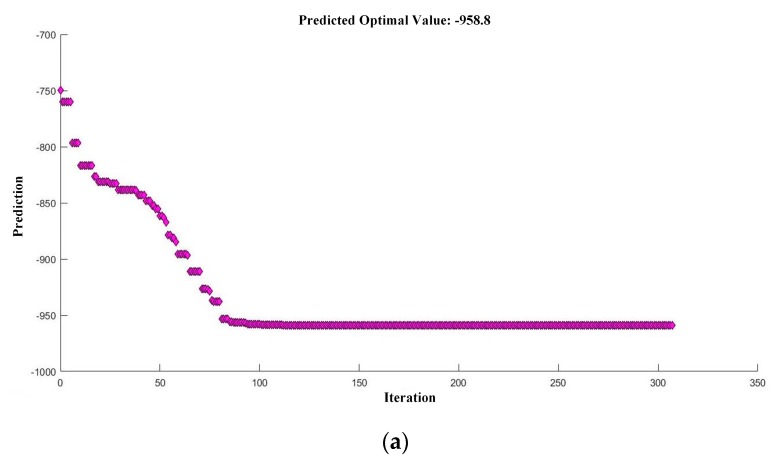

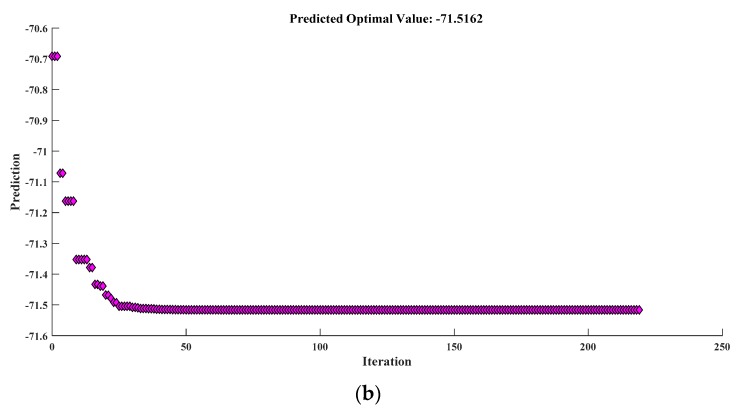
The prediction for the optimal 3D printing parameters and the corresponding flexural strength (**a**) and flexural modulus (**b**) of the printed CCF/EPCs samples.

This optimization model can be solved by various methods. It was solved by the “fmincon” function in MATLAB R2017a in this paper. The “fmincon” allows for flexibly choosing an optimization algorithm among ‘”interior-point algorithm”, “trust-region-reflective algorithm”, “sequence quadratic programming method”, and “active-set algorithm”, and the “interior-point algorithm” was employed here. The obtained optimal parameters for flexural strength were *V* = 796.9 mm/min, *H* = 0.35 mm, *S* = 1.20 mm, *T* = 166.00 °C and *P* = −99.00 kPa, and the corresponding flexural strength was 958.8 MPa (Figure 6a). The obtained optimal parameters for flexural modulus were *V* = 793.3 mm/min, *H* = 0.35 mm, *S* = 1.20 mm, *T* = 168.00 °C, and *P* = −96.00 kPa, and the corresponding flexural modulus was 71.5162 GPa (Figure 6b). To verify the optimal results, three experiments with the optimal parameters were performed (Table 3), and the average optimal flexural strength and flexural modulus were 912.1 MPa and 69.28 GPa, respectively. The relative errors of the predicted maximum flexural strength and flexural modulus were 5.1% and 3.2%, which are acceptable errors in engineering. Furthermore, since the flexural strength and flexural modulus were positively related, they should have the same optimal parameters for the maximum value. The optimal parameters for flexural strength and flexural modulus obtained by the proposed framework were very close to each other. This can cross validate each other and demonstrate the effectiveness of the proposed framework.

## 7. Conclusions

A sensitivity analysis-based optimization framework was developed to optimize the 3D printing parameters for the additive manufacturing of CCF/EPCs. The main contributions and findings are as follows.

(1) The experimental data of the additive manufacturing of CCF/EPCs were collected and validated by three-sigma rule and box-plot. All the data obtained by orthogonal experimental design and single-factor variable control method were found to be valid and, hence, used for the construction of the SVR surrogate model of the process parameter–mechanical property relationship.

(2) The variance-based SA method was adopted to analyze the influence of the 3D printing parameters (i.e., printing speed *V*, printing space *S*, printing thickness *H*, curing pressure *P*, and curing temperature *T*) on the mechanical properties (i.e., flexural strength and flexural modulus) of the printed samples. The SA found that a change in an individual input parameter had a minor effect on the variation of the flexural strength and flexural modulus which were mainly affected by the interactions among the input parameters.

(3) The optimal 3D printing parameters and the corresponding flexural strength and flexural modulus were predicted by implementing the proposed sensitivity analysis-based optimization framework. Experiments with the predicted process parameters were conducted to verify the prediction of the flexural strength and flexural modulus, and the results showed that the sensitivity analysis-based optimization framework can serve as a high-accuracy tool to optimize the 3D printing parameters for the additive manufacturing of CCF/EPCs and to predict the flexural strength and flexural modulus of the printed samples.

Essentially, the proposed sensitivity analysis-based optimization framework has universal adaptability to similar application scenarios such as process parameter optimization for automated fiber placement, data-driven bio-inspired design of strong and tough composites, etc. Normally, it is quite time-consuming and costly to acquire the experimental data, even for small datasets. Therefore, more research efforts should be devoted to the construction of a SVR surrogate model based on smaller experimental or simulation data.

It is worth noting that the sensitivity analysis-based optimization framework proposed in this paper mainly focused on the optimization and prediction of a single mechanical property. For practical problems, multiple properties are generally taken into consideration when composites are manufactured, which makes process parameter optimization very complex. Fortunately, the proposed framework can be extended to this case, where multivariate sensitivity analysis techniques and multi-objective optimization methods are required. While multivariate sensitivity analysis techniques can identify the important parameters for all the mechanical properties considered based on the SVR model of each property, the multi-objective optimization method can search the important parameters which optimize all the mechanical properties simultaneously. Further detailed research for multiple properties will be conducted in future work.

## Figures and Tables

**Table 1:** The 3D printing process parameters.

Process Parameters	Values
Printing speed (mm·min^−1^)	200~1400
Printing space (mm)	1.0~1.4
Printing thickness (mm)	0.25~0.45
Curing temperature (°C)	150~190
Curing pressure (MPa)	−0.02~−0.1

**Table 2:** Experimental data.

#	Printing Speed (mm·min^−1^)	Printing Space (mm)	Printing Thickness (mm)	Curing Temperature (°C)	Curing Pressure (MPa)	Flexural Strength (MPa)	Flexural Modulus (GPa)
1	200	1.0	0.25	150	−0.02	660.8699	57.5127
2	200	1.1	0.30	160	−0.04	717.4586	61.3590
3	200	1.2	0.35	170	−0.06	801.9369	65.4731
4	200	1.3	0.40	180	−0.08	767.1566	58.6598
5	200	1.4	0.45	190	−0.10	666.3564	54.9489
6	500	1.1	0.25	170	−0.08	861.6027	71.6292
7	500	1.2	0.30	180	−0.10	712.3392	58.8811
8	500	1.3	0.35	190	−0.02	695.9950	65.8205
9	500	1.4	0.40	150	−0.04	740.2541	57.6429
10	500	1.0	0.45	160	−0.06	842.4707	67.3847
11	800	1.2	0.25	190	−0.04	616.9910	44.3187
12	800	1.3	0.30	150	−0.06	636.2180	48.6564
13	800	1.4	0.35	160	−0.08	792.8516	63.6566
14	800	1.0	0.40	170	−0.10	697.4975	57.5773
15	800	1.1	0.45	180	−0.02	674.6801	53.1469
16	1100	1.3	0.25	160	−0.10	682.3967	51.4495
17	1100	1.4	0.30	170	−0.02	636.5848	48.9007
18	1100	1.0	0.35	180	−0.04	679.3136	50.8338
19	1100	1.1	0.40	190	−0.06	783.9741	60.8011
20	1100	1.2	0.45	150	−0.08	817.1150	66.3398
21	1400	1.4	0.25	180	−0.06	778.9613	63.7432
22	1400	1.0	0.30	190	−0.08	721.1591	57.8544
23	1400	1.1	0.35	150	−0.10	702.9702	53.3820
24	1400	1.2	0.40	160	−0.02	707.0509	53.7000
25	1400	1.3	0.45	170	−0.04	745.8622	52.9382
26	200	1.2	0.35	150	−0.10	765.1427	53.63408
27	500	1.2	0.35	150	−0.10	736.4206	53.44402
28	800	1.2	0.35	150	−0.10	746.3091	56.4504
29	1100	1.2	0.35	150	−0.10	682.7215	51.20623
30	1400	1.2	0.35	150	−0.10	634.1206	46.95916
31	800	1.2	0.25	150	−0.10	612.8753	50.9161
32	800	1.2	0.30	150	−0.10	715.8942	56.92083
33	800	1.2	0.40	150	−0.10	737.8536	52.44632
34	800	1.2	0.45	150	−0.10	742.5152	55.96409
35	800	1.0	0.35	150	−0.10	661.6145	49.49135
36	800	1.1	0.35	150	−0.10	683.9867	48.4565
37	800	1.3	0.35	150	−0.10	745.0633	58.17614
38	800	1.4	0.35	150	−0.10	821.5221	64.13514
39	800	1.2	0.35	150	−0.08	826.4611	64.51862
40	800	1.2	0.35	150	−0.06	800.9212	62.79963
41	800	1.2	0.35	150	−0.04	717.5753	62.80635
42	800	1.2	0.35	150	−0.02	648.4216	47.78122
43	800	1.2	0.35	160	−0.10	916.0076	66.69733
44	800	1.2	0.35	170	−0.10	952.8868	71.95371
45	800	1.2	0.35	180	−0.10	754.4841	61.38528
46	800	1.2	0.35	190	−0.10	720.601	51.66744

**Table 3:** The flexural strength and flexural modulus of the printed CCF/EPCs samples under the optimal parameters.

#	Flexural Strength (MPa)	Flexural Modulus (GPa)
1	897.33	69.20
2	886.15	66.70
3	952.89	71.95
